# Energy threshold changes in volcanic activity at Mt. Etna (Italy) inferred from volcanic tremor

**DOI:** 10.1038/s41598-022-20766-8

**Published:** 2022-10-25

**Authors:** Horst Langer, Susanna Falsaperla, Salvatore Spampinato, Alfio Messina

**Affiliations:** Istituto Nazionale di Geofisica e Vulcanologia, Sezione di Catania, Osservatorio Etneo, Piazza Roma 2, 95125 Catania, Italy

**Keywords:** Seismology, Volcanology

## Abstract

From the 2010s on, pattern classification has proven an effective method for flagging alerts of volcano unrest before eruptive activity at Mt. Etna, Italy. The analysis has been applied online to volcanic tremor data, and has supported the surveillance activity of the volcano that provides timely information to Civil Protection and other authorities. However, after declaring an alert, no one knows how long the volcano unrest will last and if a climactic eruptive activity will actually begin. These are critical aspects when considering the effects of a prolonged state of alert. An example of longstanding unrest is related to the Christmas Eve eruption in 2018, which was heralded by several months of almost continuous Strombolian activity. Here, we discuss the usage of thresholds to detect conditions leading to paroxysmal activity, and the challenges associated with defining such thresholds, leveraging a dataset of 52 episodes of lava fountains occurring in 2021. We were able to identify conservative settings regarding the thresholds, allowing for an early warning of impending paroxysm in almost all cases (circa 85% for the first 4 months in 2021, and over 90% for the whole year). The chosen thresholds also proved useful to predict that a paroxysmal activity was about to end. Such information provides reliable numbers for volcanologists for their assessments, based on visual information, which may not be available in bad weather or cloudy conditions.

## Introduction

**Figure 1:**
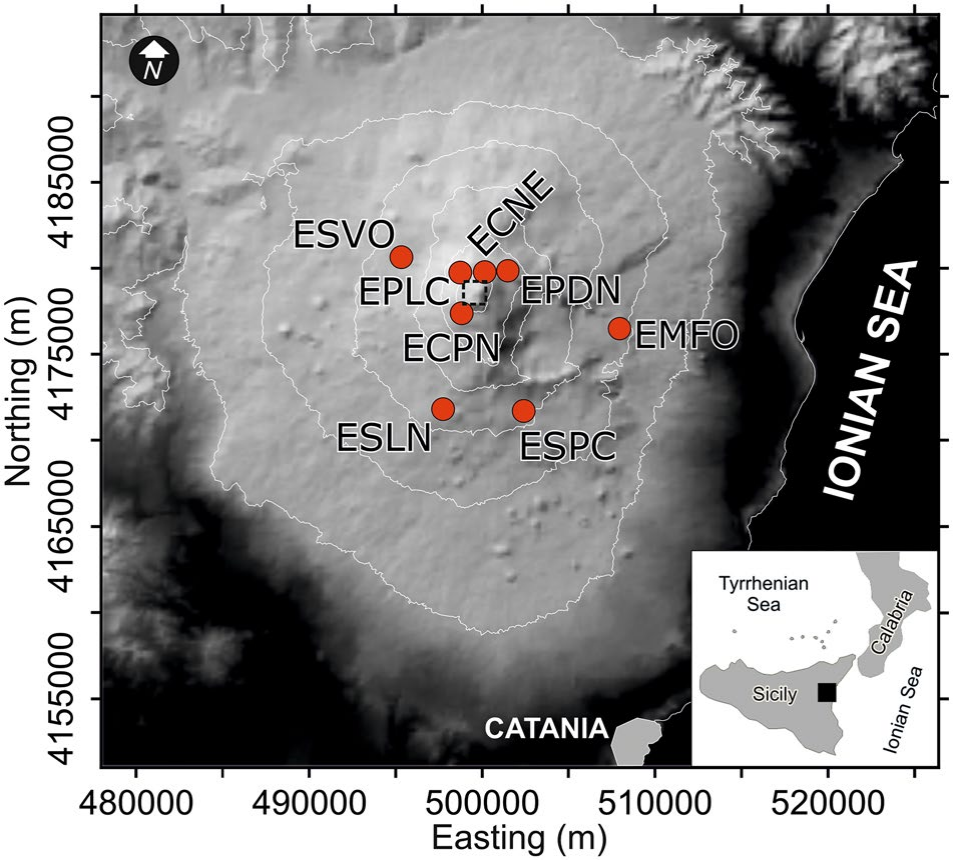
Digital elevation model (DEM) of Etna from TINITALY/01 by Tarquini et al.^26^. The DEM is referenced in the UTM WGS 84 zone 32 projection system, and is published with a CC BY 4.0 license (https://doi.org/10.13127/TINITALY/1.0; last access 14 September 2022). Red circles mark the location of the permanent seismic stations we used in this study. The dashed square marks the area of Etna’s summit craters.

Etna is an active basaltic volcano, with frequent episodes of eruptive activity in the form of Strombolian explosions, lava fountains and lava flows^1^. For example, hundreds of lava fountains occurred from 2000 to 2021 alone^2–4^. They stem from the summit craters, which are located at ~ 3300 m a.s.l., far away from inhabited areas (Fig. 1). Nevertheless, lava fountains produce abundant fallout of ash and lapilli, causing air and road traffic disruption, with heavy social and economic impacts (e.g.,^5^).

Early changes detected by a monitoring network of sensors are of paramount importance to highlight impending eruptions. In this perspective, volcano observatories around the world, such as at Kilauea (Hawaii^6^), Soufrière Hills volcano (Montserrat^7^), and Piton de la Fournaise (Réunion^8^), have not only enhanced their monitoring systems, but have also exploited cutting-edge tools for automated data processing. Unglert et al.^6^ study various unsupervised classification techniques with respect to their capacity to retrieve certain spectral patterns on Kilauea; Hammer et al.^7^ propose a dynamic application of Hidden Markov Models for event classification on Montserrat; Hibert et al.^8^ apply Random Forests to the discrimination of volcano-tectonic events from rockfalls on Piton de la Fournaise.

**Figure 2:**
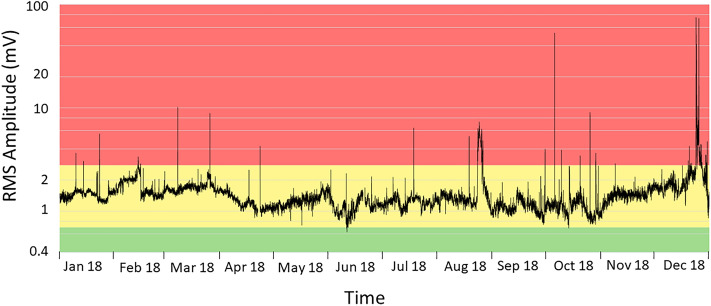
RMS amplitude of volcanic tremor at the ESLN station in 2018. The station is equipped with a velocimeter. The data are filtered between 0.5 and 2.5 Hz, and averaged over 30 min. The tremor amplitudes remained at an intermediate level, marked by the yellow band, concurrent with low to moderate Strombolian activity more or less throughout the year.

The monitoring of the background seismic radiation, which is mainly of volcanic origin and therefore called volcanic tremor (e.g.,^9^), focuses on the development over time of the amplitude and frequency content of the signal. It has become key for the surveillance of volcanic activity on Mt. Etna, where volcanic tremor is persistent^10–12^ (Fig. 1). Amplitude-ratio based criteria, such as short- and long-time averages (STA/LTA trigger algorithm), are effective in short-lived lava fountains, but have limits in the presences of a gradual increase of activity. Also, amplitude-based criteria, such as RSAM^13^, do not account for changes in the spectral characteristics, which provide information for warning purposes^14^. On Mt. Etna, the application of pattern recognition techniques to spectral features (see “Methods” section) has enabled detecting impending unrests in their very early stages (e.g.,^14^). Consequently, the staff of Istituto Nazionale di Geofisica e Vulcanologia (INGV), who runs monitoring and surveillance activity, gains precious lead time for alerting government authorities. To this end, Spampinato et al.^15^ designed a multi-station-alert system that follows the principles of voting a law in a parliament. The system counts each seismic station for which an anomaly in volcanic tremor is detected, applying triggering parameters at each single station as described by D’Agostino et al.^12^. Additional information beyond the binary decision “criticality: yes–no”, can be inferred from a voting scheme based on the number of stations where an anomaly is detected, and their weights. Even though the criticalities are flagged at each single station, the lead times vary from station to station allowing some insight into the development of an unrest. The voting essentially expresses some degree of certainty to which a criticality is declared (here, the detection of specific variations in the frequency content of the signal radiated by the volcanic system). However, a condition of volcano unrest may hold true for months and not just be short-lived. This is a serious drawback even during time spans with mild, long-lasting volcanic activity, as the voting scheme does not explicitly account for the signal energy at each station. A similar condition, hovering around low to moderate Strombolian activity, occurred at Etna more or less over the whole of 2018. Tremor amplitudes remained at an intermediate level, which is marked by a yellow band in Fig. 2. As a consequence, many stations maintained an alert flag over long-time spans before a climactic eruption started on Christmas Eve. Multidisciplinary studies have actually documented prolonged magma replenishment from depth starting at least 6 months before the eruption (e.g.,^16^).

The concept to flag even modest signs of unrest was successful for the previous years, starting from the 2010s^14^; however, the almost continuous state of alert in 2018 jeopardized confidence in the robustness of the system as if it were due to a malfunction. To overcome the drawback, we envisaged the use of thresholds of the flag alert not only for the detection of the unrest (such as weak to mild Strombolian activity in the context of Etna), but also for the onset of impending, (more energetic) eruptive activity.

**Figure 3:**
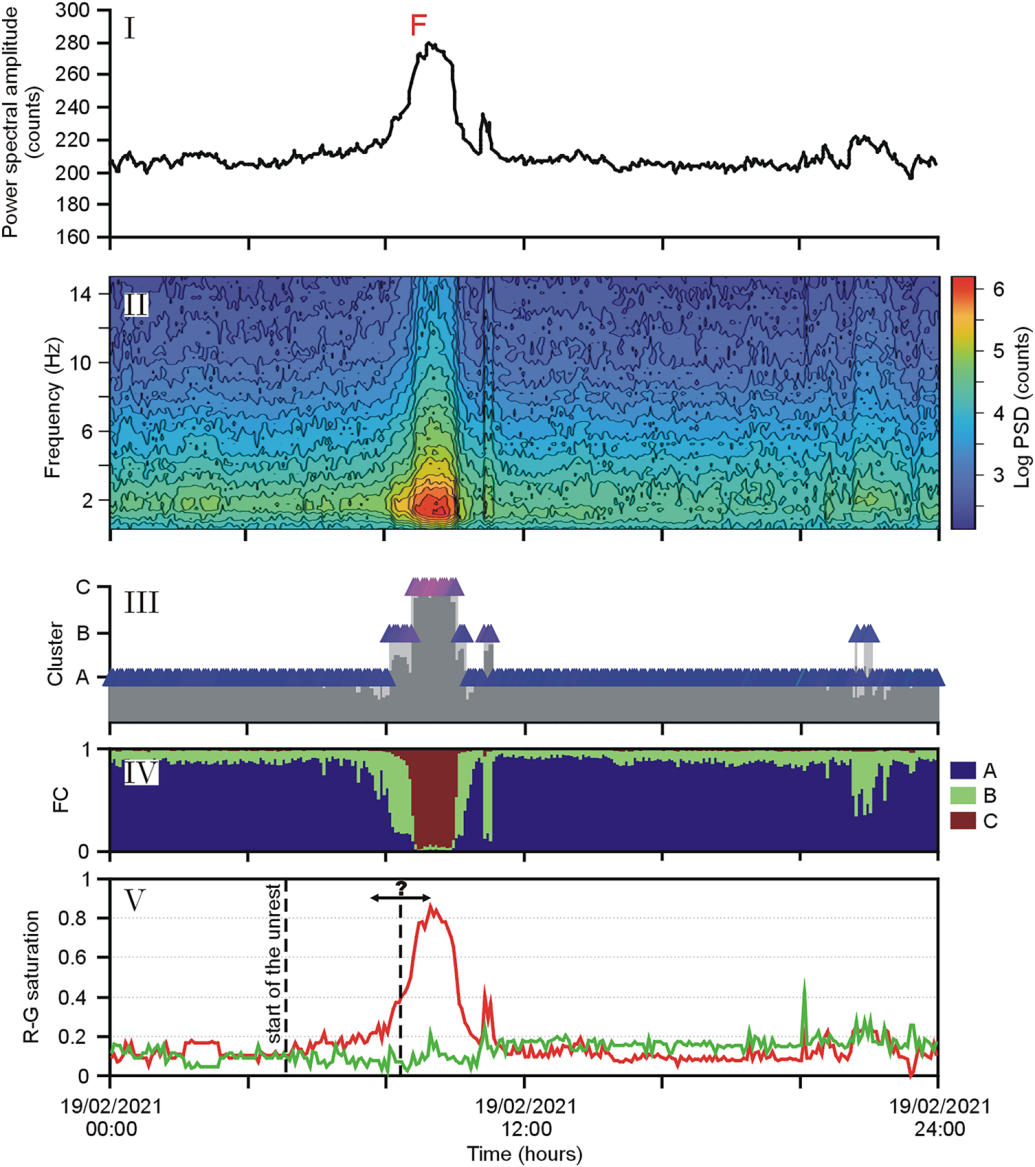
Episode of lava fountain (F) at Etna on February 19, 2021. Based on the analysis of volcanic tremor at the ESVO station, the diagrams cover 24 h: (**I**) spectral amplitude; (**II**) spectrogram; (**III**) results of the SOM in the form of colored triangles; (**IV**) fuzzy clustering (FC) considering three clusters; (**V**) normalized values of the saturation of the Red-Green (R-G) components in the SOM colors. The first dashed line marks the start of the unrest (warning flag of the multi-station system by Spampinato et al.^15^). The second dashed line (with the question mark) refers to the possible range of transition to a paroxysmal activity.

Figure 3 refers to a lava fountain episode on February 19, 2021. In the figure, we depict the spectral amplitude of volcanic tremor (panel I); the spectrogram with frequency content up to 15 Hz (panel II); and the results from Self-Organizing Maps (SOM) (panel III) and fuzzy clustering (FC) (panel IV) where the membership of a pattern to a cluster is given by a membership vector (see "Methods" section). We exploit the possibility given by the SOM to represent the spectral characteristics as an RGB (Red–Green–Blue; see "Methods" section) color code, which allows us to visualize the development of pattern characteristics over time as a sequence of colored symbols. A simplified, yet effective representation consists in plotting the saturation degrees of the Red-Green components (R-G saturation in panel V of Fig. 3) in the SOM colors. The two R-G saturation curves can be easily used to define thresholds and trigger criteria, and we focus on their variation in the following sections.

The spectral amplitudes peaked at ~ 9:00 UTC (the climax of the lava fountain) and then sharply decreased, so that at 10:15 UTC the phenomenon was essentially over (Fig. 3I–II). The development of the spectral characteristics is clearly mirrored in the SOM colors (Fig. 3III). Before the onset of the eruption, the symbols had typically blue colors that changed to purple and intense red as the paroxysmal phase approached. The climax of the lava fountain had a clear regime during which cluster “C” showed up in the SOM (Fig. 3III). The presence of this cluster has been reported as typical for eruptive activity with intense tremor radiation^14,15^. In the R-G curves, the saturation of the red component was also achieved during the climax of the volcanic activity, when the spectral amplitudes peaked (Fig. 3V). We noticed that the trend of the R saturation (R hereafter) shown in Fig. 3 initially had a slow increase, which speeded up only when the paroxysm became impending. From a visual inspection of those values, a ‘critical’ threshold of R could be inferred somewhere between 0.55 or 0.6, which marked a sharp acceleration towards the value 1 (full red saturation) shortly after. We observed that such a trend also holds for the R-G values of the lava fountains in 2011 from Spampinato et al.^15^ (see Supplementary Information). Moving on from this evidence, we investigated the presence of ‘critical’ thresholds of the R-G values, leading eruptive activity towards a climax. Here, we focus on two time-intervals, namely the year 2018 with the December 24 climactic lava effusion, and the time span from January to April 1, 2021 with 19 paroxysmal lava fountains. The results of our analysis were then tested on a new dataset encompassing all the 33 lava fountain episodes that occurred from May to December 2021.

### Fixing the threshold

**Figure 4:**
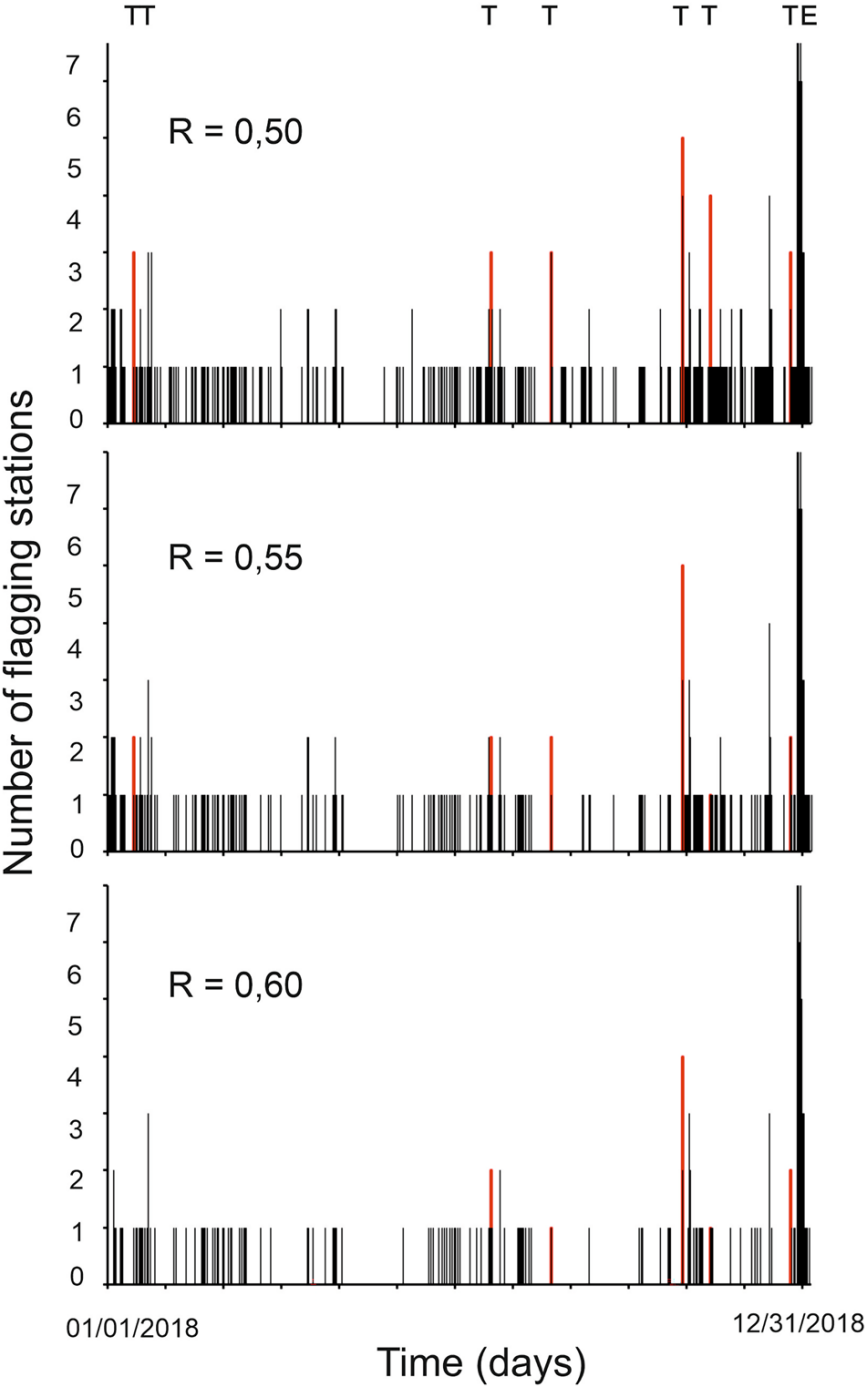
Number of stations that reached or topped three arbitrary threshold values (0.50, 0.55, and 0.60) of the R saturation in 2018. T and E stand for teleseisms and the Christmas Eve eruption, respectively. Red bars mark warnings caused by teleseisms.

The Christmas Eve eruption in 2018 was heralded by short-term (days) as well as long-term (months) changes in ground deformation, tectonic seismicity, and magma composition (e.g.,^16–18^). In Fig. 4 we show how many stations reached or topped three arbitrary threshold values of the R saturation, namely 0.5, 0.55 and 0.6, in 2018. Discarding peaks associated with the occurrence of earthquakes (mostly regional events or teleseisms marked with T in Fig. 4), we notice that R = 0.6 was reached at four stations on December 9. Only a lower number of stations reached synchronously this value from January to December 9, a timeframe during which volcanologists reported Strombolian activity and a few overflows at the summit craters^4^. On the other hand, all stations topped the value 0.6 ~ 2 ½ hours before the Christmas Eve eruption on December 24, and even peaked full saturation (R = 1) during the climax of the eruption.

So far, we have considered thresholds, such as 0.5, 0.55 or 0.6, for the R component moving on from rather generic considerations on Fig. 3, the Supplementary Information, and somewhat following our intuition. However, each decision made on the basis of the information provided by volcanic observatories comes with costs and efforts. In this context, it is mandatory that criteria are defined following reproducible rules. Any rule for setting up criteria must deal with a principal dilemma: The greater the degree of confidence in our system, the more challenging the choice of thresholds and criteria will be. The major challenge comes from the risk of losing relevant eruptive activity for some reasons, such as peculiarities of the phenomenon or temporary technical failures of the seismic network. In other words, we have to appraise various criteria comparing the “true positives” (event correctly flagged) with respect to “false positives” (event flagged without having occurred). The so-called ‘Receiver Operation Characteristic’ (ROC hereafter) curves^19^ are often used in binary decision problems, such as business decisions (e.g., launching a marketing campaign) or health care issues (for instance, whether to start a certain medical treatment). Here they allow us to establish rules for the identification of suitable criteria to evaluate the performance of our system. In ROC diagrams, we plot for each criterion the ‘True Positive Rate’ (TPR; the share of recognized events with respect to all those truly occurred) versus the ‘False Positive Rate’ (FPR), which is obtained from the ratio of all erroneously identified events with respect to all observations without the event of interest.

In the following, we discuss how to set up the threshold in the saturation of the red component (R) and the number of stations for which we require that the threshold is reached or topped in 2021. The year 2021 was mostly characterized by frequent episodes of paroxysmal volcanic activity, mainly lava fountains preceded and followed by longer time intervals with Strombolian activity and sporadic episodes of ash emission and lava effusion. The lava fountains were short-lived events with durations of several hours^20^. Their column height reached up to 10 km above ground, and formed a considerable threat to the air traffic for the fallout of pyroclastic material and ash over the municipalities located along the flanks of the volcano^4^.

Here, we start considering the time span from January 1st to April 1st, 2021, during which there were 19 lava fountains along with almost persistent Strombolian activity. To select the threshold, we consider five alternatives of the R value ranging from 0.5 to 0.6, counting the number of stations where the chosen saturation value is reached or topped. Our observations are evaluated every 5 min. This time span corresponds to the one for which the patterns, represented by the colored symbols, are obtained. Consequently, we have 288 evaluations per day, in total 26,208 patterns in the considered time span.

**Figure 5:**
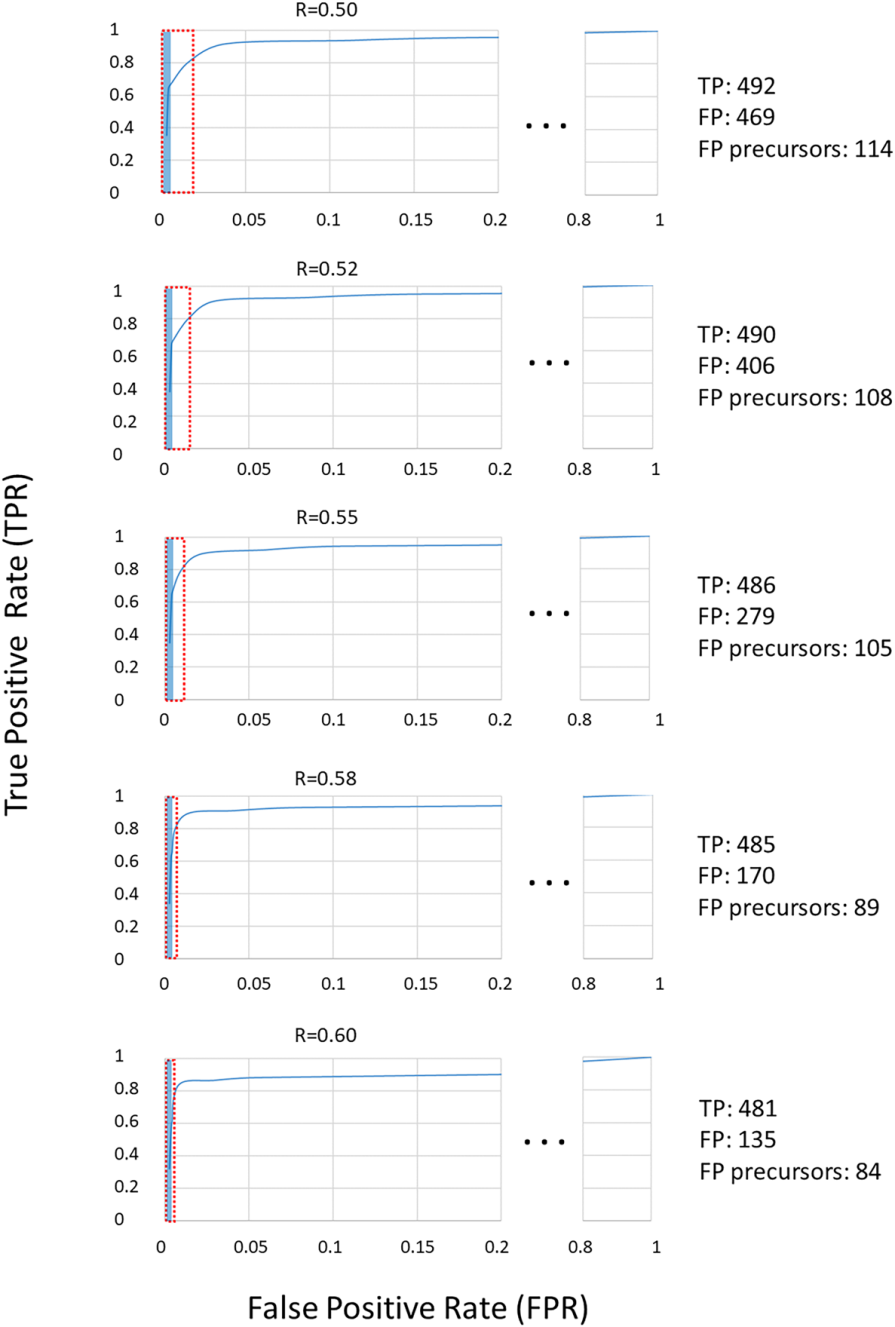
Receiver Operation Characteristic (ROC) curves for various threshold values of the R saturation. The number of True Positives (TP) and False Positives (FP) refers to a warning with at least four stations. The red and blue rectangles mark the range of FP for each threshold and the part of FP that comes as a precursor, respectively (the corresponding values are shown on the right side of the figure).

Keeping the threshold fixed, we count how many true and false positives (TP and FP) were declared accounting for the number of stations reaching that threshold. Figure 5 depicts the ROC curves obtained for R equal to 0.5, 0.52, 0.55, 0.58, and 0.6. Seven stations were functioning in the time span from January 1st to April 1st (Fig. 1), with occasional gaps at single stations. On the whole, there were 635 time intervals of 5 min concurrent with a paroxysm, which marked the highest value reachable for true positives, namely TPR = 1. In terms of the maximum false positive rate FPR = 1, we acquire a number of 25,573 (5-min) time intervals, whereas TPR = 1 is never reached. Figure 5 shows the ROC curves for various threshold values of the R saturation, with their major variations in the range 0 ≥ FPR ≤ 0.2.

**Figure 6:**
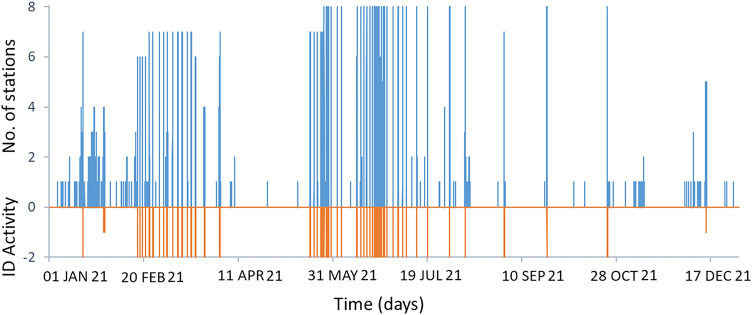
Number of stations reaching the threshold R = 0.58 (blue line, positive values) and volcanic activity (orange line) in 2021. The negative values in the y-axis are associated with the presence of strong effusive activity with ash emission (“− 1”) and lava fountains (“− 2”), respectively.

The interpretation of ROC curves can be guided by the so-called ‘Area Under the Curve’ (AUC), which is essentially the integral of an ROC curve. In case of a mere random result, then 0.5 < AUC < 0.6, namely TPR and FPR increase to the same degree, and the diagonal line across the ROC diagram has slope 1. The performance is good when 0.8 < AUC < 0.9^15,19^. Overall, all ROC curves shown in Fig. 5 have AUC ~ 0.95, a value indicative of an excellent performance of the system. The calculation of these AUCs, however, is ill-conditioned here, due to problems arising from the low number of TP (635) with respect to the maximum number of possible FP (i.e., ca. 26,000). This leads to errors in computing the integral of the ROC. Indeed, the tail of the ROC with FPR greater than 0.1 dominates the AUC, even though being supported by only one FPR/TPR pair. As mentioned above, we have no valid configuration achieving TPR = 1. Our discussion will therefore focus on the absolute values of TP and FP (see Table 1). In general, we get TPR ~ 0.8 (namely ~ 480 out of 635 true positives for all ROC) when requiring that the threshold for an alert flag is reached in at least 4 stations. In this case, the number of false positives is 135 for R = 0.6, 170 for R = 0.58 and reaches 469 when choosing R = 0.50. Based on the values in Table 1, a configuration with a minimum of four flagging stations (called “Min4”) and thresholds such as R = 0.58 or 0.6 keeps the number of false positives well below the number of true positives (Fig. 6). With less conservative options (R = 0.5 and R = 0.52), more or less equal numbers of true and false positives are obtained.

**Table 1:** True and False Positives (TP and FP) for various thresholds of the R value and number of stations flagging an alert.

Stations	FP	TP	FP	TP	FP	TP	FP	TP	FP	TP
0	25,573	635	25,573	635	25,573	635	25,573	635	25,573	635
1	5352	580	4202	578	2951	573	2280	564	1398	560
2	3129	569	2359	565	1674	559	1147	552	741	549
3	1086	557	854	553	622	545	413	544	269	542
4	469	492	406	490	279	486	170	485	135	481
5	155	406	136	405	120	400	117	394	112	390
6	112	387	108	386	108	381	101	379	100	375
7	84	214	81	213	79	211	74	206	68	203
Thresh	R = 0.50		R = 0.52		R = 0.55		R = 0.58		R = 0.60

Requiring a rate close to 90% of detected true positives, we can opt for three flagging stations and all thresholds from R = 0.5 to R = 0.6. However, with the less conservative choices (R = 0.5 and R = 0.52) we encounter a considerable amount of FP (twice the number of TP for R = 0.5 and 1.5 fold the number of TP for R = 0.52). Again, the conservative choices may be preferred.

### Lead times

In many applications, for instance in medical disciplines, false positives are generally undesired. They can be the ruin of disease diagnostics, as the chance of belonging to a group of erroneously positive subjects tops the risk of being among the people actually affected by a disease. In those cases, at least a second test is recommended (e.g.,^21^). Such a rigid understanding of false positives is not feasible for our problem. In the context of eruption forecasting, volcanic observatories are interested in precursory phenomena, that is, peculiarities which may herald the unrest. Such precursors are intrinsically false positives, even though they are welcome for the needs of ‘lead times’, namely the timeframe between the moment when the first positive (precursor) is detected and the true positive is observed.

To be accepted as precursors in our system, we require that the positives detected form a chain longer than 5 min without gaps (no more than one positive missing in the sequence). As an example, we list in Table 2 the time difference between when the value 0.58 of the R component was reached and the moment in which the onset of each of the paroxysmal events from January to April 1, 2021 was reported (see also Fig. 6). Here the lead time is positive when the threshold R = 0.58 was reached before the onset given by the INGV timely communications to government authorities, such as Civil Protection, prefectures, and aviation authorities. As patterns are created every 5 min and the precision of the times reported is even less, the lead times of Table 2 are multiples of 5 min. In most cases, lead times are positive and vary from a few minutes to several hours depending on the dynamics of the eruptive event. The ECNE station, being one of the closest to the summit craters where the activity started, is the first to signal it at almost any time. On the other hand, the station ESVO is in most cases the last site where the threshold R = 0.58 was reached. The “Max” column in Table 2 shows the longest lead times among all operating stations, which are obtained requesting that only one station meets the threshold. The highest value (255 min, corresponding to 51 patterns) refers to the station ESPC for the paroxysm on April 1, 2021, whereas ECNE had on average the largest lead times. In fact, if we sum up all lead times (keeping the positives only), we obtain 213 patterns that fall in the lead time range, namely the patterns that can be considered as precursors (see row “Sum/5” in Table 3). Nonetheless, there are 2280 false positives for the same configuration (Table 1). In a more conservative configuration, where we request that the threshold is met at four or more stations, we still obtain positive lead times in 15 out of the 19 reported paroxysms (see Table 2, column “Min4”); in one case (“nd” in the table) the number of operating stations was less than four.

**Table 2:** Lead times per station. Column “Max” is the maximum lead time encountered. Column “Min4” corresponds to a lead time requesting a minimum of four triggering stations. Values are obtained for a threshold R = 0.58.

Station/Episode	ECNE	ECPN	EMFO	EPLC	ESLN	ESPC	ESVO	Max	Min4
1: 18/01/2021 20:00–21:05	50	− 10	40	10	10	15	− 5	50	10
2: 16/02/2021 16:10–17:00	10	− 5	0		0	0	− 5	10	0
3: 18/02/2021 00:00–00:50	10	0	15		5	20	5	20	5
4: 19/02/2021 08:50–09:50	20	0	15		15	20	5	20	15
5: 20/02/2021 22:00–01:15	30	− 50	25		− 5	− 35	− 30	30	− 30
6: 22/02/2021 22:25–00:15	45	− 30	35	35	5	− 25	− 15	45	5
7: 24/02/2021 19:30–22:30	45	5	10	5	15	5	5	45	5
8: 28/02/2021 07:40–08:20	0	− 15	− 10	− 10	− 5	− 10	− 15	0	− 10
9: 02/03/2021 12:20–14:50	10	− 30	− 5	5	5	5	− 5	10	5
10: 04/03/2021 02:20–03:15	20		− 20					20	Nd
11: 04/03/2021 07:50–09:25	60	0	5	5	30	5	0	60	5
12: 07/03/2021 06:00–07:10	90	40	65	40	85	30	35	90	40
13: 10/03/2021 00:40–03:30	120	65	85	90	100	95	75	120	90
14: 12/03/2021 07:40–09:55	65	25	30	60	45	30	30	65	30
15: 14/03/2021 23:40–02:40	65	40	50	60	70	60	40	70	60
16: 17/03/2021 02:15–06:15	45	25	35	50	45	40	30	50	40
17: 19/03/2021 08:35–10:20	55	45	50		50	50	40	55	50
18: 23/03/2021 22:00–08:45			35		50	40	45	50	35
19: 01/04/2021 00:00–08:05	235	55	35		245	255	50	255	50
Median	45	0	30	35	22.5	20	5		
Average value	54.17	9.41	26.05	31.82	42.50	33.33	15.83

**Table 3:** Comparison of lead times (given in minutes) for various thresholds of the R value for the 19 lava fountain episodes from January 1 to April 1, 2021. Sum/5 is the sum of the lead times expressed in terms of the number of the patterns, each of which covers 5 min.

Episode	Max	Min4	Max	Min4	Max	Min4	Max	Min4	Max	Min4
1	325	15	325	15	80	15	50	10	40	5
2	20	0	15	0	10	0	10	0	5	0
3	85	5	75	5	20	5	20	5	20	10
4	35	15	35	15	20	15	20	15	20	10
5	45	− 20	45	− 25	30	− 30	30	− 30	30	− 30
6	85	5	65	5	65	5	45	5	40	5
7	55	10	55	10	55	5	45	5	15	5
8	0	− 5	0	− 5	0	− 10	0	− 10	− 5	− 10
9	25	5	25	5	10	5	10	5	5	− 5
10	55	nd	55	nd	25	nd	20	nd	− 10	nd
11	100	15	95	10	95	5	60	5	60	5
12	145	85	135	85	105	85	90	40	85	40
13	165	95	165	100	165	100	120	90	110	90
14	115	40	75	40	70	40	65	30	60	35
15	100	60	100	60	70	60	70	60	65	55
16	60	55	60	45	60	45	50	40	50	40
17	70	50	70	50	65	50	55	50	55	40
18	60	55	50	35	45	35	50	35	50	30
19	335	60	310	60	255	55	255	50	255	50
Sum/5	376	114	351	108	249	105	213	89	193	84
Thresh	R = 0.50		R = 0.52		R = 0.55		R = 0.58		R = 0.60	

Table 3 gives a summary of the lead times for various thresholds of the R value, namely 0.50, 0.52, 0.55, 0.58, and 0.60. Keeping R = 0.58 and increasing the minimum number of alert flags to four stations (Fig. 6), the resulting lead time is the value reported in the column “Min4” in Table 3. As before, if we sum up the lead times in that column (keeping the positives only), we obtain 89 positive patterns as precursors. Comparing the 89 precursor patterns to the total of 170 false positives for the configuration R = 0.58 / four stations (see Table 1), we find this configuration more convenient than the previous choice with only one station. Focusing on the “Max” and “Min4” columns of this table, we find that also the configuration R = 0.60 / four stations provides a reasonable performance. With this configuration we have a total of 135 FP (see Table 1), 84 of which are actually precursors (Table 3).

In the choice of the most convenient configuration of our system, we may also consider the appearance of cluster C instead of the saturation in red. Cluster C shows up in the context of strong volcanic activity^14^ (see panel III in Fig. 3). If we calculate the lead times replacing the R threshold with patterns prevalently belonging to cluster C, we obtain a similar picture as before (see Table 4). Again, in the majority of cases, the ECNE station is the one where cluster C appears first, and ESVO is typically the last one. The values in the “Max” and “Min4” columns resemble those found for R = 0.52 or 0.55 in Table 3. Consequently, from a practical viewpoint, it may be sufficient to focus on the R thresholds, as the appearance of the cluster C does not really add new, independent evidence. Cluster C also appears due to sources which are not linked to the volcano, such as during teleseisms or strong stormy weather (see, e.g.,^14^).

**Table 4:** Lead times calculated from the appearance of cluster C.

Station/Episode	ECNE	ECPN	EMFO	EPLC	ESLN	ESPC	ESVO	Max	Min4
1: 18/01/2021 20:00–21:05	active	− 5	40	0	25	20	10	40	10
2: 16/02/2021 16:10–17:00	10	− 5	0		0	0	0	10	0
3: 18/02/2021 00:00–00:50	15	5	15		10	25	5	25	15
4: 19/02/2021 08:50–09:50	20	5	30		20	20	5	30	20
5: 20/02/2021 22:00–01:15	30	− 45	40		30	− 20	− 5	40	30
6: 22/02/2021 22:25–00:15	45	− 5	35	30	30	0	30	45	30
7: 24/02/2021 19:30–22:30	35	10	10	5	20	10	5	35	10
8: 28/02/2021 07:40–08:20	− 5	− 10	− 5	− 10	− 5	− 5	− 10	− 5	− 5
9: 02/03/2021 12:20–14:50	10	0	5	0	5	5	0	10	5
10: 04/03/2021 02:20–03:15	20		15					20	Nd
11: 04/03/2021 07:50–09:25	70	5	15	5	45	15	15	70	15
12: 07/03/2021 06:00–07:10	90	65	85	45	95	80	60	95	80
13: 10/03/2021 00:40–03:30	120	85	95	110	110	105	85	120	105
14: 12/03/2021 07:40–09:55	65	40	40	60	65	40	40	65	40
15: 14/03/2021 23:40–02:40	65	60	50	55	75	70	50	75	60
16: 17/03/2021 02:15–06:15	50	30	45	50	75	50	30	75	50
17: 19/03/2021 08:35–10:20	55	50	50		55	50	45	55	50
18: 23/03/2021 22:00–08:45			60		15	50	55	60	15
19: 01/04/2021 00:00–08:05	250	200	65		255	265	60	265	60
Sum/5								227	119
Median (min)	45	7.5	37.5	37.5	30	25	30		
Average value (min)	55.58	30.63	36.11	35	52.94	44.71	27.65		

## Discussion and conclusions

In this paper, we calculate the ‘true’ value of thresholds at Etna that may lead to a climax after the detection of volcanic unrest. To this end, we have refined a previous alert system by Spampinato et al.^15^ based on a multi-station voting scheme. This kind of voting provides information redundancy, which improves robustness against possible operational failures of a single station—a frequent phenomenon in harsh environmental conditions. A further point is the increased reliability of an alert issued from a group of stations, which is less affected by local disturbances, such as environmental noise or instrumental effects. However, after the scheme sums over all the positive votes and issues an alert, there is no indication of the intensity of the impending eruptive activity. The decision-based vote may also be insufficient when moderate anomalies become persistent over long-time spans. In the year 2018, for instance, the volcano fed Strombolian activity of low to modest intensity over several months, yielding a nearly uninterrupted alert flag. Such a continuous, longstanding switched-on red light may decrease the staff’s level of attention in a volcano observatory and can even be annoying for the authorities, who operate on an emergency basis. It is therefore important to mark ‘points of no return’, i.e., thresholds which, once topped, herald an impending change to a major increase of the eruptive activity. Analyzing the Supplementary Information (redrawn from Spampinato et al.^15^), we surmised that such thresholds may indeed exist. Convincing evidence for their existence has been found from a visual inspection of the R-G curves, in which the red component (R = 0.6 or higher values) in the SOM appeared as a good candidate. We also tried to figure out at how many stations the threshold has to be reached or topped. In a new, in-depth analysis we focused on the period from Jan 1st to April 1st, 2021, when 19 lava fountains occurred. This data set has been used as a learning ensemble for the choice of a threshold, marking the non-return point, and the number of stations to consider. The choice was guided using Receiver Operation Characteristic (ROC) curves, which come as diagrams of true-positive vs. false-positive rates. In these diagrams we count, for each specific threshold and number of stations where the threshold is met, how many times a critical phenomenon—here a paroxysmal event—was truly identified by the spectral characteristics of the patterns (true positives; TP). We compare these TP to the number of cases where the phenomenon was flagged, without actually having occurred. A commonly used parameter describing the validity of a ROC curve is the Area Under the Curve (AUC). Unfortunately, the calculation of AUC is ill-conditioned in our context, as we have no configuration where we are able to recognize all true positives, even requiring that the threshold is reached at only one station. However, this should not be taken as a deficiency. In fact, a part of the ‘false positives’ belongs to the so-called lead time, the time interval leading to the occurrence of the phenomenon we are interested in. Those false positives are in reality precursors, thus welcome in the context of monitoring and alert. We carried out a systematic analysis of true and false positives, varying both the threshold of the red saturation (R = 0.5…0.6), as well as the number of stations where this should be reached or topped. We found a reasonable compromise for R = 0.58 or 0.6 together with the request that this R value should be reached at least in four stations (Fig. 6). In these configurations, a majority of the false positives fall into lead times. In other words, they are precursors rather than errors caused by noise or other problems.

Applying the same scheme, that is, considering saturation thresholds of the red component and a (minimum) number of stations, we essentially cancel out the phenomenon of a continuous alert we were facing before. Certainly, the volcano was in an almost persistent state of unrest in the year 2018 until December, when the climactic Christmas Eve eruption occurred. In Fig. 4 we recognize that, with the requirement of at least 3 stations reaching a threshold of R = 0.5, we acquire some false alarms mainly caused by the presence of regional earthquakes and teleseisms due to their low frequency content. Using a more conservative setting, i.e. R = 0.55 or higher, and requiring at least 4 stations to reach the threshold, we actually get no more than one false alarm, which occurred on October 25, 2018 during a teleseism.

To test if the system is up to the task with the chosen thresholds, we considered the 33 episodes of lava fountains from May to December 2021. We focused on the two conservative settings mentioned above, i.e. R = 0.58 or R = 0.6, always requiring the threshold to be reached or topped at a minimum of four stations. In both the configurations we almost always noticed positive lead times, i.e., the impending paroxysmal activity was detected before its occurrence was reported in the communications released by INGV-OE. In only two single cases was the detection delayed by 5 min (1 pattern length). On average, the lead times were ~ 40 min with R = 0.58 and ~ 35 min choosing R = 0.6. A further issue is the ‘closure’ of the activity, i.e., the prediction that the eruptive crisis is going to end. In both configurations, the closure was declared, on average, 20 min earlier than the end times reported in the communications (see Table 5). Occasionally, the closure by our revised system was delayed, producing a number of false positives. However, these were essentially the only false positives encountered in the tested period May-December, 2021.

**Table 5:** Average lead and closure time for the lava fountain episodes from May to December 2021. “Closure” reports the time difference between the declared end and the actual warning of the episodes.

	Lead Time R = 0.58	Closure	Lead Time R = 0.6	Closure
Average (min)	40.6	20.5	34.5	20
Median (min)	30	15	25	15

In a recent paper published during the preparation of the present study, Calvari and Nunnari^20^ re-analyzed the visual and infrared- images recorded by the INGV video cameras installed on the volcano. They considered 66 lava fountains occurring from December 2020 to February 2022, for each of which they report start and end times inferred from their analysis of the images. We decided to use their study as a further test of our system. We compared the times they report for the eruptive episodes in 2021 with the times we calculated, considering the configuration R = 0.58 and four or more stations reaching or topping this threshold. Table 6 gives a summary of the comparison. On average, our system had an additional positive lead time of ~ 13 min (the corresponding median is 8 min). The absolute discrepancies (counting negative and positive differences, all positive) are somewhat higher. On the other hand, end times were fairly close to each other. The tremor-based system marked the end of an episode on average 6.8 min later (median is 0); taking the absolute differences we obtain an average of 10 min (median 5 min).

**Table 6:** Comparison between the times calculated by Calvari and Nunnari, 2022 (CN22 in the table) for the eruptive episodes in 2021 and those we obtained with the threshold R = 0.58 and four or more flagging stations.

	Difference start R = 0.58 − CN22	Difference closure R = 0.58 − CN22
Average (min)	13.0	− 6.9
Median (min)	8	0
Average abs. Difference (min)	29.8	10.5
Median abs. Difference (min)	25	5

The comparison of our results to those inferred by the detailed analysis of the video camera images highlights that the tremor-based system is a valid proxy for the automatic identification of impending paroxysmal events. Note that two of the episodes reported in 2021 by Calvari and Nunnari^20^, namely those on February 23 at 03:45 UTC and March 4 at 01:30 UTC, were among the weakest in terms of the ‘Time Averaged Discharge Rate’ given by the authors. Those episodes did not reach the energy levels of tremor needed to trigger the alert flag at four stations. As the transition of various states of volcanic activity is not always clear cut, the tremor-based criteria may provide a guide to help volcanologists to better distinguish the activity regimes.

We can therefore envisage using a two-stage warning system. The first, which has been operating so far remains active, as it effectively recognizes mild phenomena of unrest, such as Strombolian activity, or minor lava effusions, not always leading to a relevant threat to human facilities. In the presence of a first stage of warning, the observatory staff will follow the ongoing development. Once the second level of criticality is reached (e.g., R = 0.58 at four stations or more) little doubt is left that the situation is heading towards a larger unrest, such as a paroxysmal lava fountain or a major lava emission as happened during the Christmas Eve eruption in 2018. It is worth noting that such a two-stage warning system overcomes problems of methods based on STA/LTA strategies, which fail when there is a slow increase in energy of the seismic radiation. Indeed, in this case, LTA slowly creeps to high values, which cannot be easily topped by the STA.

The practical choice of the parameters R and the number of stations is partly a matter of the decision makers, who decide whether the actual risk of missing an event can be accepted, and what is the desired lead time. A comparison of the number of patterns, being precursors with respect to the total of false positives encountered for a configuration, can guide the choice. Here, using R = 0.58 or 0.6 and four stations, most of the false positives come as precursors, thus they are opportune rather than representing a deficiency of the system. Others occur shortly after a paroxysmal phenomenon dies out, as their occurrence is still linked to the fading volcanic activity.

## Methods

### Feature extraction

Langer et al.^14^ developed a scheme for automatic feature extraction based on spectral analysis, which resembles the Seismic Spectral Amplitude Measurement (SSAM) proposed by Rogers and Stephen^22^. The first step is the calculation of the Short Time Fourier Transform (STFT), with a gliding window applied to the whole time series. Each window has a length of 1024 points (corresponding to 10.24 s) and is shifted by 500 points. Each short-time spectrum forms an element in a spectrogram. Frequency bins are averaged over the power spectral amplitudes in a selected frequency band to reduce the number of features. A further simplification is obtained by considering an ensemble of 60 short-time power spectra, i.e., a time span of 5 min. We preferred to study the 10% (bottom) percentile, focusing on the lowest amplitudes encountered in the 5-min time span, to largely eliminate the effect of short-lived transients (e.g., wind gusts or local earthquakes).

### Self-organizing maps

SOM—or Kohonen maps^23^—are a type of artificial neural network. SOM form a mesh of nodes, which are small clusters with each one representing a number of patterns. The centroid of the clusters can be understood as a prototype of patterns. During the learning phase, the feature vectors W of the centroids are adjusted so that the sum of the distances between the original data and their representing prototype nodes converges to a minimum. Formally, during the training of SOM, one minimizes the sum of the distances

$$D_{ij} = \sqrt {({\mathbf{W}}_{i} - {\mathbf{V}}_{j} )^{T} ({\mathbf{W}}_{i} - {\mathbf{V}}_{j} )}$$

where Vj is the normalized input feature vector and Wi the weights stored in the nodes. V and W have the same dimension. A core step is the identification of the closest node to the actual input vector, i.e., the best matching unit (BMU) for the jth pattern. During the learning steps, neighboring nodes lying within a certain radius of influence are considered as well. Weights are gradually adjusted according to the so-called learning rate λ, which decreases with time t. A second parameter φ (called influence radius) describes the dependence on distance ∆ of the upgrade of a node. φ (∆,t) is maximum for the BMU, whereas nodes outside the radius of influence are not upgraded at all. Similar to the learning rate λ, also φ (∆,t) decreases with time. The upgrade of weights follows the relationship:

$${\mathbf{W}}_{i} (t + 1) = {\mathbf{W}}_{i} (t) + \varphi (\Delta ,t) \cdot \lambda (t) \cdot D_{ij} (t)$$

The introduction of the term φ (∆,t) has an important effect, known as ‘Topological fidelity’, i.e. patterns represented by neighboring nodes in the SOM are truly close to each other also in the original data space. SOM can be particularly instructive when the weights take the form of a color code. For this purpose, Principal Component Analysis is applied, projecting the multi-dimensional weights vector in a 2D representation space spanned by the principal axes z1 and z2. The coordinates of a node with respect to the axes are expressed in terms of a color code, such that saturation in red (“R”) stands for the first, saturation in green (“G”) for the second axis, and saturation in blue is just complementary to saturation in green. The coding allows identifying the position of a pattern on the map simply by an appropriate colored symbol. For more details, the reader can consult textbooks such as Kohonen^23^ or Langer et al.^24^.

Spampinato et al.^15^ probed Self-Organizing Maps (SOM) in the framework of a multi-station system aimed at early warning. They considered continuously recorded data from 11 permanent seismic stations. The seismic network was composed of two rings with the center at the summit craters: an inner ring of seven stations (within a radius of ∼3 km), and an outer ring with four stations placed at distances of up to ~ 8 km (Fig. 1). Following the strategy proposed in Spampinato et al.^15^, our trigger parameters are defined for each single station. These parameters are basically based on the RGB color codes, with “R” (red) and “G” (green) being independent, whereas “B” (blue) is complementary to “G”, i.e., B = 1 − G. The classifier has been tested on past data streams, encompassing various episodes of eruptive activity for which each of the 11 seismic stations issued a timely warning.

### Fuzzy clustering

The cluster analysis follows a non-hierarchical, partitioning strategy of clustering. The measure of the heterogeneity of the k-th cluster is expressed as the sum of the squared distances of the patterns in each cluster from their centroid vector. Contrary to crisp clustering, where each pattern is exclusively assigned to one cluster, in fuzzy clustering^25^ each pattern may belong to a certain degree to all possible classes. Consequently, the class membership of a pattern, rather than being a simple ID, is given by a vector. The partition is consequently described by a M × K matrix of class membership values, with M being the number of patterns in the entire data set, and K the number of clusters. Transitional regimes become evident in the gradual changes in the class membership vector. In our application, we calculate the fuzzy membership vectors on the original feature vectors and show them together with the SOM colors. In Fig. 3 (see panel IV) the patterns are assigned to the cluster with the highest membership degree.

## Supplementary Information

Supplementary Information.

## Data Availability

The raw seismic data analysed during the current study are available in the European Integrated Data Archives (www.eida.ingv.it). The software for the pattern recognition can be freely downloaded from https://earthref.org/ERDA/974/. The dataset that we used to create the statistics of this article are freely available in https://doi.org/10.13127/etna/mavt2021.
