# Supplementary material for: Energy threshold changes in volcanic activity at Mt. Etna (Italy) inferred from volcanic tremor
Source: Sci Rep. 2022 Oct 25;12:17895. doi: 10.1038/s41598-022-20766-8 (PMC9596467; doi:10.1038/s41598-022-20766-8)
Supplement: Supplementary file 1 — Supplementary Information. [file 41598_2022_20766_MOESM1_ESM.pdf]

Supplementary information

for

Energy threshold changes in volcanic activity at Mt. Etna (Italy) inferred from volcanic tremor

Horst Langer, Susanna Falsaperla, Salvatore Spampinato, Alfio Messina*

Istituto Nazionale di Geofisica e Vulcanologia, Sezione di Catania, Osservatorio Etneo, Piazza Roma 2, 95125, Catania, Italy

***Corresponding Author**:

Susanna Falsaperla
Istituto Nazionale di Geofisica e Vulcanologia, Sezione di Catania
Piazza Roma 2, 95125, Catania, Italy
tel. number 39-095-7165845
e-mail: susanna.falsaperla@ingv.it

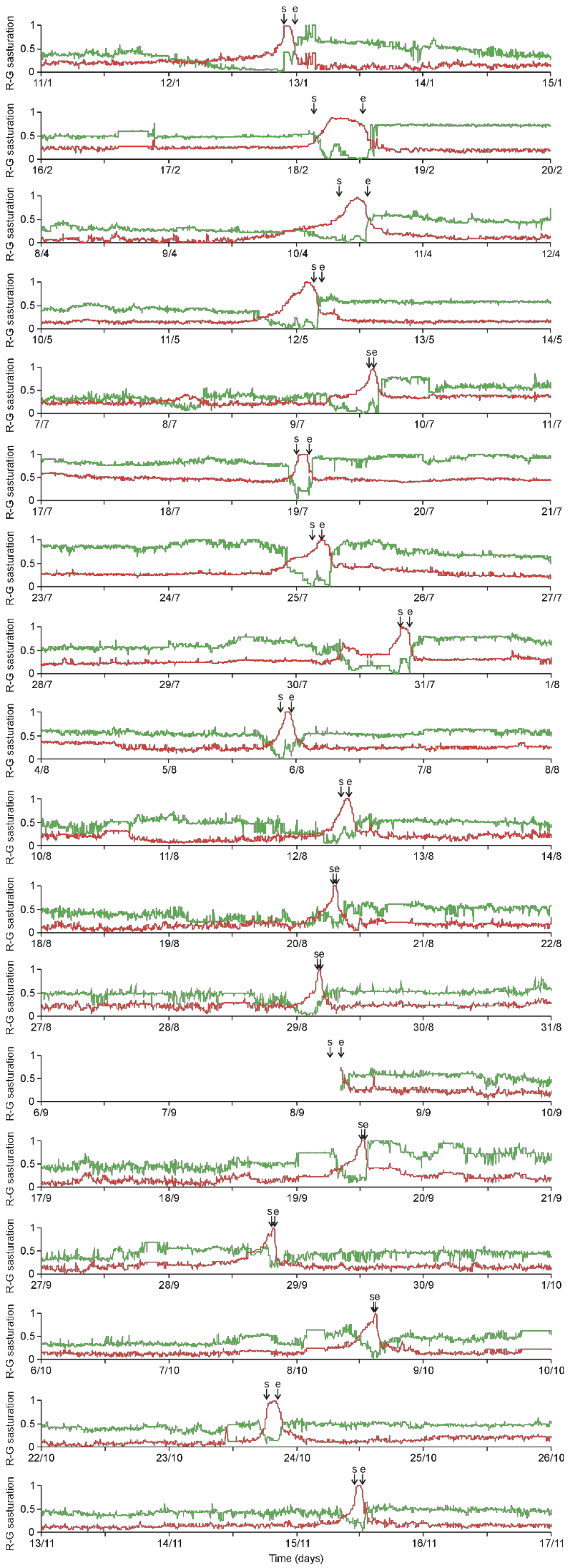
R-G sasturation
1
0.5
0
s e
11/1 12/1 13/1 14/1 15/1
16/2 17/2 18/2 19/2 20/2
8/4 9/4 10/4 11/4 12/4
10/5 11/5 12/5 13/5 14/5
7/7 8/7 9/7 10/7 11/7
17/7 18/7 19/7 20/7 21/7
23/7 24/7 25/7 26/7 27/7
28/7 29/7 30/7 31/7 1/8
4/8 5/8 6/8 7/8 8/8
10/8 11/8 12/8 13/8 14/8
18/8 19/8 20/8 21/8 22/8
27/8 28/8 29/8 30/8 31/8
6/9 7/9 8/9 9/9 10/9
17/9 18/9 19/9 20/9 21/9
27/9 28/9 29/9 30/9 1/10
6/10 7/10 8/10 9/10 10/10
22/10 23/10 24/10 25/10 26/10
13/11 14/11 15/11 16/11 17/11
Time (days)

Figure 1S - Normalized values at the ECPN station of the red (R) and green (G) components in the RGB color code of the SOM results for the 18 lava fountain episodes in 2011. The arrows mark onset (s) and end (e) of each lava fountain as documented by Behncke et al.[1] (modified from Spampinato et al.[2]).

References

[1] Behncke, B. et al. The 2011–2012 summit activity of Mount Etna: Birth, growth and products of the new SE crater. J. Volcanol. Geotherm. Res. 270, 10–21 (2014).

[2] Spampinato, S. et al. Short-term detection of volcanic unrest at Mt. Etna by means of a multi-station warning system. Scientific Reports, 9:6506, https://doi.org/10.1038/s41598-019-42930-3 (2019).
